# The Influence of Methylsulfonylmethane on Inflammation-Associated Cytokine Release before and following Strenuous Exercise

**DOI:** 10.1155/2016/7498359

**Published:** 2016-10-23

**Authors:** Mariè van der Merwe, Richard J. Bloomer

**Affiliations:** School of Health Studies, The University of Memphis, Memphis, TN, USA

## Abstract

*Background*. Inflammation is associated with strenuous exercise and methylsulfonylmethane (MSM) has been shown to have anti-inflammatory properties.* Methods*. Physically active men were supplemented with either placebo or MSM (3 grams per day) for 28 days before performing 100 repetitions of eccentric knee extension exercise.* Ex vivo *and* in vitro* testing consisted of evaluating cytokine production in blood (whole blood and isolated peripheral blood mononuclear cells (PBMCs)) exposed to lipopolysaccharide (LPS), before and through 72 hours after exercise, while* in vivo* testing included the evaluation of cytokines before and through 72 hours after exercise.* Results*. LPS stimulation of whole blood after MSM supplementation resulted in decreased induction of IL-1*β*, with no effect on IL-6, TNF-*α*, or IL-8. After exercise, there was a reduced response to LPS in the placebo, but MSM resulted in robust release of IL-6 and TNF-*α*. A small decrease in resting levels of proinflammatory cytokines was noted with MSM, while an acute postexercise increase in IL-10 was observed with MSM.* Conclusion*. Strenuous exercise causes a robust inflammatory reaction that precludes the cells from efficiently responding to additional stimuli. MSM appears to dampen the release of inflammatory molecules in response to exercise, resulting in a less incendiary environment, allowing cells to still have the capacity to mount an appropriate response to an additional stimulus after exercise.

## 1. Introduction

Inflammation is a normal biological response to harmful stimuli, including pathogens and sterile tissue damage. The response originates with innate immune cells that sense danger through pattern recognition receptors (PRR) such as Toll-like receptor 4 (TLR4) and respond by producing molecules to initiate the inflammatory cascade that eventually leads to pathogen clearance and tissue repair [1]. This cascade of events is tightly controlled, as chronic inflammation has deleterious consequences and is associated with a plethora of diseases, including cardiovascular disease [2] and rheumatoid arthritis [3].

Methylsulfonylmethane (MSM) is an organosulfur compound that is widely used as a dietary supplement for inflammatory conditions such as osteoarthritis [4] and allergic rhinitis [5].* In vitro* studies indicate that the anti-inflammatory activity of this compound is mediated by the inhibition of the proinflammatory nuclear factor kappa beta (NF-*κβ*) signaling pathway and attenuation of the NLR family pyrin domain containing 3 (NLRP3) inflammasome activation, resulting in decreased release of the proinflammatory cytokines such as interleukins IL-1*β*, IL-6, and IL-8 [6–8]. In addition to pathological conditions, MSM supplementation also alleviates markers of oxidative stress and muscle damage following acute bouts of exercise in a healthy population [9–12]. However, as strenuous exercise also induces an inflammatory response, it is not known what effect MSM has on the inflammatory cytokine production.

Considering the available evidence, we hypothesized that supplementation with MSM would reduce* in vivo* inflammatory cytokine production induced by a single bout of strenuous eccentric exercise—exercise which is known to induce an acute inflammatory response [13, 14]. In order to test this hypothesis, we analyzed human blood samples for cytokine production. Specifically, blood from healthy exercise-trained men was collected before and through 72 hours following the completion of an acute bout of strenuous eccentric exercise. In addition to* in vivo* cytokine analysis, blood samples were also stimulated* ex vivo* and* in vitro* with lipopolysaccharide (LPS) to determine changes in cytokine production induced by a Toll-like receptor 4 (TLR4) agonist.

## 2. Methods

### 2.1. Subjects

A total of 40 men began the study and completed testing. Subjects were healthy, physically active, and nonsmokers and did not have any cardiovascular or metabolic disorders. All subjects were required to have been performing resistance exercise for a minimum of six months prior to beginning the study, with at least one day per week of lower body resistance exercise. They were not using nutritional supplements or medications that may have impacted inflammatory status. If subjects were noted upon screening to be using supplements or medications thought to impact outcome measures, they were required to cease use for at least three weeks prior to the start of the study. Subject characteristics are provided in Table 1. As can be seen, the mean body mass index is greater than 25 kg/m^2^. However, due to the exercise training status of subjects, many were muscular and carried more body mass because of this. By no means were these subjects carrying a significant amount of body fat. Subjects were informed of all procedures, potential risks, and benefits associated with the study, the procedures were approved by the University Institutional Review Board for Human Subjects Research, and subjects provided written informed consent.

### 2.2. Supplementation

This study involved a double-blind, placebo-controlled design in which a total of 40 subjects completed testing (*in vivo* portion only). Subjects were assigned to MSM (*n* = 20; 3 grams per day; OptiMSM®, Bergstrom Nutrition, Vancouver, WA) or placebo (*n* = 20; rice flour) capsules to ingest daily for four weeks, in addition to the three days following the exercise bout. The rice flour placebo has been used in many prior studies and noted to be inert. Compliance to intake was determined by counting capsules upon bottle return. Subjects assigned to each condition were matched based on age and body mass in an effort to retain similarity between the MSM and placebo groups.

### 2.3. Exercise Bout and Collection of Blood Samples

Subjects performed 10 sets of 10 repetitions (or repetitions to failure) of eccentric knee extension using roughly 100% of concentric one-repetition maximum, with a 2-minute rest period between sets. During the exercise, the weight on the apparatus was raised by the investigators to a level where the knees were fully extended. The subject then lowered the weight under control on a 4-second count (using a metronome) to the ending position. If the subject was unable to lower the weight over a 4-second count, the weight was reduced by approximately 10% for the remaining sets to maintain the repetitions at 10 per set. Several days prior to performing the actual exercise protocol, subjects were familiarized with the knee extension apparatus and performed dynamic repetitions in an attempt to prepare for the actual test session.

Before (Baseline 1) and following the 4-week supplementation period (Baseline 2), as well as at 0, 24, 48, and 72 hours after exercise, a venous blood sample was obtained from a forearm vein. Whole blood collected into ethylenediaminetetraacetic acid (EDTA) tubes was removed and treated accordingly for* ex vivo* and* in vitro* analysis (*n* = 2, placebo; *n* = 3, MSM). Blood collected into tubes containing no additive was allowed to clot at room temperature for 30 minutes and was then centrifuged to obtain serum and stored at −70°C until analyzed for cytokines.

### 2.4. *Ex Vivo* Analysis

As a pilot experiment and using a sample of only 5 of the 40 subjects, blood was collected and analyzed. For whole blood LPS stimulation, 250 *μ*L of whole blood was incubated in a 96-well U-bottom plate with 50 *μ*L culture media (RPMI 1640, Pen/Strep, and 10% fetal bovine serum (FBS)) containing LPS to a final concentration of 0.2 *μ*g/mL. For unstimulated control samples, 50 *μ*L of culture media was added to 250 *μ*L of whole blood. All samples were incubated at 37°C for 24 h in a humidified environment containing 5% CO_2_. After incubation, supernatants were collected after centrifugation and stored at −70°C.

Cytokine concentrations were measured using a Millipore multiplex magnetic bead system and a MAGPIX® analyzer. Antibodies for the following analytes were used: IL-1*β*, IL-17a, TNF-*α*, IL-8, IFN-*γ*, IL-10, IL-6, and MCP-1.

### 2.5. *In Vitro* Analysis

Peripheral blood mononuclear cells (PBMCs) were isolated using Ficoll (Ficoll-Paque PLUS, GE Healthcare Life Sciences) according to manufacturer's instructions. Cell number was determined and cells were diluted in culture media (RPMI 1640, Pen/Strep, and 10% FBS). 300,000 cells were plated per well in a 96-well U-bottom plate. LPS was added to a final concentration of 0.2 *μ*g/mL. As an unstimulated control, cells were also incubated in culture media in the absence of LPS. Cells were incubated at 37°C for 24 h in a humidified environment containing 5% CO_2_. After incubation, the supernatant was collected and stored at −70°C.

Cytokine concentrations were measured using a Millipore multiplex magnetic bead system and a MAGPIX analyzer. Antibodies for the following analytes were used: IL-1*β*, IL-17a, TNF-*α*, IL-8, IFN-*γ*, IL-10, IL-6, and MCP-1.

### 2.6. *In Vivo* Analysis

Whole blood was analyzed for cytokines using a MILLIPLEX MAP human custom cytokine magnetic bead panel including analytes for the following cytokines: IL-10, IL-17a, IL-1*β*, Il-6, IL-8, and TNF-*α* (EMD Millipore; HCYTOMAG-60K). Serum was used without dilution according to the manufacturer's instructions and analytes were quantified using MAGPIX and xPONENT software. All assays were performed in duplicate on the first thaw.

### 2.7. Statistical Analysis

For* in vivo* testing, cytokines were analyzed using repeated measures analysis of variance (RMANOVA), using data obtained from preexercise (Baseline 2 only) and all postexercise times. Tukey* post hoc* tests were used as needed. Baseline 1 and Baseline 2 values were compared using analysis of variance. Statistical significance for all tests was set at *p* ≤ 0.05.

For* ex vivo* and* in vitro* analysis, statistical significance was not determined, as there were only *n* = 2 (placebo) and *n* = 3 (MSM). Mean ± SEM was calculated using GraphPad Prism 6.

## 3. Results

### 3.1. *Ex Vivo*

**Figure 1:**
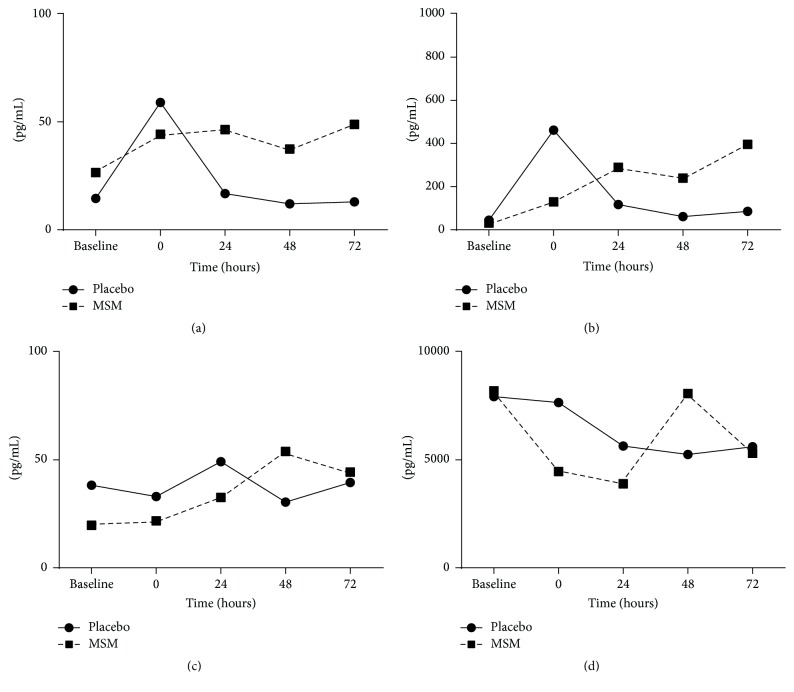
Reduced induction of inflammatory cytokines with MSM supplementation immediately after exercise. IL-*β* (a), IL-6 (b), TNF-*α* (c), and IL-8 (d) concentrations (pg/mL) were determined in whole blood* ex vivo* cultures from blood collected at baseline (24 h before exercise), immediately after exercise (Time 0), and then at 24 h, 48 h, and 72 h after exercise. Results represent mean (*n* = 2, placebo; *n* = 3, MSM).

Exercise is a physiological stress that results in systemic alterations in hormones and cytokines and also affects leukocyte functionality and survival [15]. To determine the role of MSM in exercise-induced changes in leukocytes response, whole blood cultures (where blood was collected before and after exercise) were incubated with or without LPS and secreted cytokine levels determined. In the absence of MSM and LPS, exercise induced an acute increase in IL-1*β* and IL-6, with maximum response immediately after exercise (time = 0) and a return to baseline at 24 h. However, in the presence of MSM, this exercise-induced response is dampened immediately after exercise but appears to increase at later time points (24 h and 48 h after exercise) (Figures 1(a) and 1(b)). In addition, TNF-*α* and IL-8 appear to have a delayed cytokine response with maximum production at 48 h after exercise (Figures 1(c) and 1(d)).

**Figure 2:**
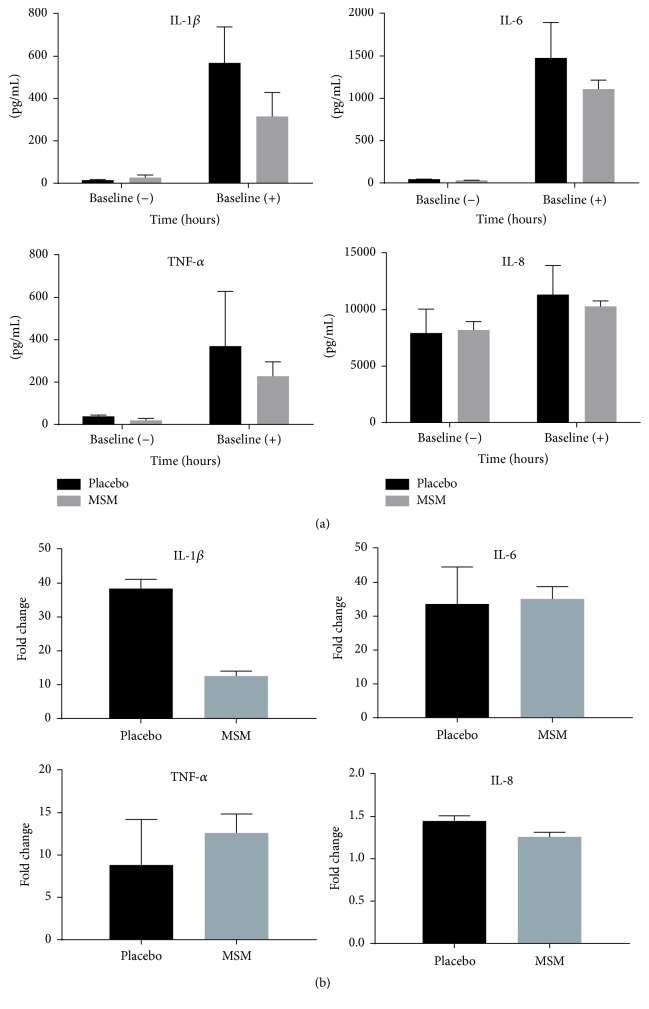
MSM reduces LPS-induced IL-1*β* secretion. Cytokine secretion was determined after LPS stimulation of* ex vivo* whole blood cultures and measured by Luminex multiplex bead assay. (a) In the absence of exercise, LPS induces robust release of IL-1*β*, IL-6, TNF-*α*, and IL-8. (b) Data from (a) are expressed as fold change from unstimulated samples. Results represent mean ± SEM (*n* = 2, placebo; *n* = 3, MSM).

In these experiments, LPS is used as a stimulus for* ex vivo* whole blood cultures to determine responsiveness of leukocytes present in the blood before and after exercise in the presence or absence of MSM. As whole blood is used for this experiment, MSM would be present in the soluble fraction. Incubation of whole blood (collected before exercise, baseline) with LPS induced robust release of IL-1*β*, IL-6, TNF-*α*, and IL-8 (Figures 2(a)-2(b)). This is consistent with LPS being a TLR4 agonist and inducing inflammatory cytokine synthesis and release. To normalize for cytokine levels present in blood plasma without LPS addition, fold changes in cytokine levels were calculated. MSM supplementation very efficiently suppressed IL-1*β* induction but was unable to suppress IL-6, TNF-*α*, and IL-8 to the same extent (Figure 2(b)).

**Figure 3:**
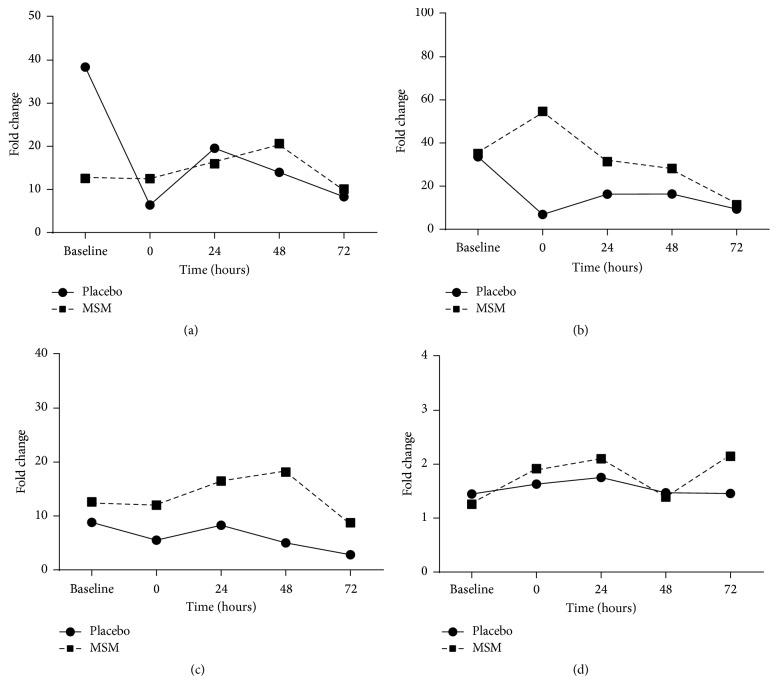
Exercise alters cytokine response to LPS. IL-1*β* (a), IL-6 (b), TNF-*α* (c), and IL-8 (d) induced by LPS, with MSM supplementation allowing for more efficient release of IL-6 and TNF-*α* after exercise. Data are represented as fold change from unstimulated samples. Results represent mean ± SEM (*n* = 2, placebo; *n* = 3, MSM).

We next determined the effect of MSM supplementation on LPS-induced cytokine levels after exercise from whole blood collected immediately after (time 0) and 24 h, 48 h, and 72 h hours after exercise (Figure 3). In the placebo group, there was a dramatic reduction in LPS-induced IL-1*β* and IL-6 secretion immediately after exercise (Figures 3(a) and 3(b)). Surprisingly, MSM appears to protect against this loss with IL-1*β* levels comparable to baseline and an increase in IL-6 secretion immediately after exercise in the MSM supplemented group (MSM* versus* placebo; 3104.233 pg/mL ± 1036.124* versus* 1334.280 pg/mL ± 926.401). Neither exercise nor MSM appeared to influence the levels of LPS-induced TNF-*α* and IL-8 (Figures 3(c) and 3(d)).

To determine whether this induction is specific to IL-1*β* and IL-6, we also determined the effect of LPS on additional cytokines associated with tissue damage and inflammation. LPS did not induce the secretion of IL-17*α*, IFN-*γ*, and MCP-1 (data not shown).

**Figure 4:**
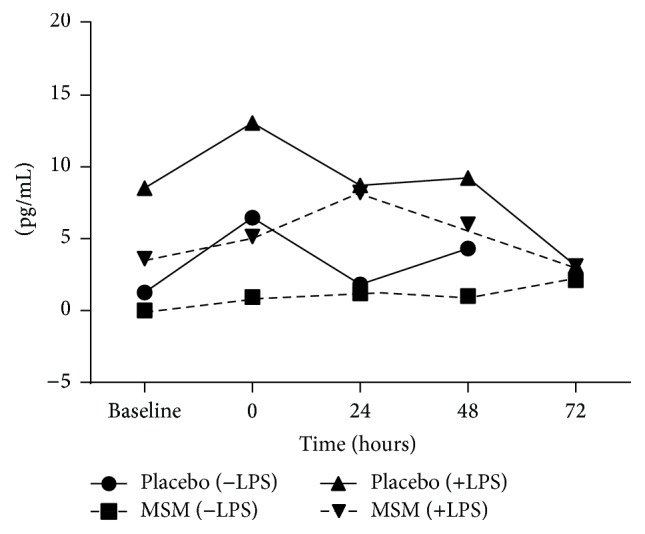
Induction of IL-10 by exercise and LPS in* ex vivo* cultures. Results represent mean ± SEM (*n* = 2, placebo; *n* = 3, MSM).

IL-10 is a cytokine that can repress proinflammatory responses and limit unnecessary tissue damage. In whole blood samples incubated for 24 h in the absence of LPS, IL-10 levels were very low/undetectable (Figure 4). Similar to what was seen for IL-6, the placebo group had an increase in IL-10 immediately after exercise as seen in samples not stimulated by LPS (Figure 4 (−LPS)). This is consistent with the fact that both pro- and anti-inflammatory molecules are released in response to exercise with the goal of inducing and then resolving inflammation. With LPS stimulation of these samples, IL-10 is induced to the same extent for both groups, with a greater absolute increase in the MSM supplemented group at 24 hours after exercise (Figure 4, MSM (+LPS)).

### 3.2. *In Vitro*

**Figure 5:**
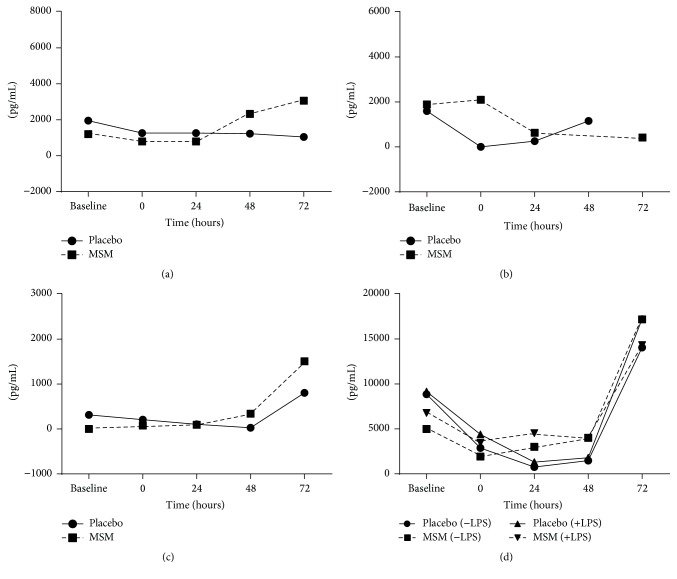
IL-1*β* (a), IL-6 (b), TNF-*α* (c), and IL-10 (d) release from* in vitro* LPS-stimulated PBMCs isolated from blood collected before (baseline) or after (0 h, 24 h, 48 h, and 72 h) exercise. Results represent mean ± SEM (*n* = 2, placebo; *n* = 3, MSM).

To control for confounding factors present in the soluble fraction of blood and also to normalize for cell numbers, PBMCs were isolated from blood collected at time points mentioned earlier.* In vitro* cultures of PBMCs were corrected for cell number and incubated in tissue culture media in the presence or absence of LPS for 24 h. In addition to the loss of soluble factors, MSM would also not be present in these cultures. In cultures of PBMCs isolated from blood collected before exercise, LPS efficiently induced the secretion of IL-1*β*, IL-6, and TNF-*α* as seen in Figures 5(a)–5(c). Contradictory to the results from the* ex vivo* cultures, LPS-induced IL-1*β* secretion in preexercise cultures is more similar between the placebo and the MSM groups (Figure 5(a)), suggesting that there is a loss of the anti-inflammatory capability when culturing in tissue culture media. This result further implies that MSM present in the soluble fraction is responsible for inhibition of LPS-induced IL-1*β* secretion. In addition, cultured isolated PBMCs did not have the dramatic loss of LPS-induced cytokines immediately after exercise (Figures 5(a) and 5(b)), suggesting that, rather than loss of function, there is loss of leukocytes with exercise resulting in reduced cytokine release. Interestingly, IL-6 levels were still robustly induced with LPS in the MSM supplemented group immediately after exercise (Figure 5(b)). This is consistent with what is seen in the whole blood incubation, with IL-6 secretion not being suppressed by MSM but rather augmented by exercise. There was no difference between the placebo and MSM supplemented groups for TNF-*α* (Figure 5(c)), IL-8 (data not shown), and IL-10 (Figure 5(d)).

### 3.3. *In Vivo*

For the* in vivo* testing, the following was noted: no condition (*p* = 0.42), time (*p* = 0.99), or interaction (*p* = 0.91) effect was noted for IL-6. No condition (*p* = 0.19), time (*p* = 0.71), or interaction (*p* = 0.97) effect was noted for IL-8. A condition effect was noted for IL-10 (*p* = 0.001), with values higher for MSM compared to placebo. No time effect (*p* = 0.96) or interaction effect (*p* = 0.95) was noted. A condition effect was noted for IL-17 (*p* = 0.0005), with values lower for MSM compared to placebo. No time effect (*p* = 0.64) or interaction effect (*p* = 0.77) was noted. A condition effect was noted for IL-1*β* (*p* = 0.0003), with values higher for MSM compared to placebo. No time effect (*p* = 0.92) or interaction effect (*p* = 0.91) was noted. A condition effect was noted for TNF-*α* (*p* < 0.0001), with values higher for MSM compared to placebo. A trend for a time effect was noted (*p* = 0.08). No interaction effect was noted (*p* = 0.95). Data are presented in Table 2.

## 4. Discussion 

In the current study, we determined the effect of MSM supplementation on cytokine release in response to exercise and how it alters the response of leukocytes to an additional stimulus in the form of LPS. Blood was collected from healthy subjects before and after exercise. Cytokine release was determined* ex vivo*, where whole blood was exposed to LPS;* in vitro*, isolated PBMCs exposed to LPS; and* in vivo*, where systemic cytokine levels were determined for each subject at different time points after exercise.

Consistent with previous data suggesting that MSM acts as an antioxidant [9–11], MSM was able to dampen the acute induction of IL-1*β* and IL-6 to an intense bout of exercise. Using LPS as a second stimulus, whole blood collected before exercise was stimulated and could very efficiently induce IL-1*β*, IL-6, TNF-*α*, and IL-8. However, MSM supplementation specifically inhibited the secretion of IL-1*β* and possibly IL-6 and TNF-*α*, but to a lesser extent. This reduction is also consistent with previous* in vitro* results demonstrating that MSM blocked IL-1*β* secretion from LPS-primed bone marrow-derived macrophages [6].

To determine the effect of MSM on exercise-induced changes in leukocytes, blood was also collected after exercise and stimulated with LPS to determine whether MSM supplementation alters the immune cell response after a stressor such as exercise. The acute bout of exercise resulted in a dramatic reduction in LPS-induced IL-1*β*, IL-6, and TNF-*α* after exercise in the placebo group. Interestingly, MSM supplementation protected against this loss and the LPS-induced response was similar to baseline levels for the MSM group. This immunosuppressed phenotype can be a consequence of changes in the soluble fraction of the blood, including differences in hormones or additional cytokine that was not measured. Intense bouts of exercise can also cause a decrease in TLR4 expression, decreasing response potential of the leukocytes [16]. In addition, it might also result from changes in leukocyte number due to redistribution or apoptosis, as previous studies [17] have shown that intense exercise leads to leukopenia, depending on exercise and intensity.

As we did not determine leukocyte number in the* ex vivo* cultures, PBMCs were isolated and equal cell numbers stimulated with LPS to see whether the results mimic the* ex vivo* cultures. These cultures were incubated in tissue culture media thereby removing any incongruent factors that might be present in the soluble fraction of the whole blood culture. IL-1*β* was again very efficiently induced by LPS in the PBMC cultures. Contradictory to the* ex vivo* cultures, cells from the MSM group responded similarly to the placebo group. As MSM is not present in the PBMC cultures (still present in the plasma in the* ex vivo* cultures), our data suggests that MSM is responsible for the inhibitory effect on IL-1*β*. However, we cannot rule out the fact that there are additional molecules in the soluble fraction that might influence the IL-1*β* release.

Of great importance is the fact that the PBMC cultures did not have a significant difference between the placebo and MSM group immediately after exercise, as seen with the* ex vivo* cultures, where there was a dramatic loss of responsiveness to LPS in the placebo group. This suggests that the decrease in LPS-induced cytokines in the placebo group reflects reduction in cell number. As there is no decrease from baseline to time 0 for the MSM group in* ex vivo* cultures, our data also suggests that MSM is protecting against exercise-induced leukopenia. Interestingly, LPS-induced IL-6 induction was as efficiently induced in* ex vivo* cultures as isolated PBMCs, indicating that the leukocytes have been primed previously to release IL-6 by the exercise-induced environment and that MSM does not inhibit this release of IL-6. IL-6 can act as a myokine and a proinflammatory cytokine and might be beneficial when released after a bout of tissue damaging exercise.

*In vivo* inflammatory markers were not different between MSM and placebo, with a great deal of variability across subjects in most measures (Table 2). While not statistically significant, MSM treatment tended to reduce resting proinflammatory cytokines (e.g., IL-6, IL-8, and IL-17) when viewing the change from Baseline 1 to Baseline 2, while these values increased with placebo treatment. In response to exercise, proinflammatory cytokines increased and gradually decreased from Baseline 2 through the postexercise period with a similar pattern in both groups. An anti-inflammatory cytokine (IL-10) increased immediately after eccentric exercise in the MSM group (51%) and decreased slightly in the placebo group. It was reported that IL-10 is increased in response to exercise which induces muscle damage [18] and this can act as an anti-inflammatory mediator by downregulating proinflammatory cytokines such as IL-1*β* and TNF-*α* [19]. The present study noted that IL-10 levels were correspondingly elevated after exercise, possibly to compensate for damage-induced increase in proinflammatory cytokines. This trend occurred only in the MSM group but not in the placebo group. Based on our observation, while only speculative, MSM supplementation may improve resting systemic inflammatory status while simultaneously sensitizing the acute anti-inflammatory response to eccentric exercise.

It should be noted that inflammation was only analyzed in blood samples. It is possible that inflammation may have been elevated in skeletal muscle. Our failure to include skeletal muscle samples for analysis may be considered a limitation of this work.

## 5. Conclusions

Taken together, these data suggest that an intense bout of exercise causes muscle damage that induces a robust inflammatory reaction that results in exercise-induced leukocyte death and temporary immunosuppression. MSM, acting as an antioxidant, is able to blunt tissue damage and the resulting inflammation and, as a consequence, prevent leukocyte apoptosis. As inflammation induced by exercise may cause prolonged muscle soreness, these data are in agreement with the results noting a reduction in muscle soreness after exercise with MSM supplementation [11]. Based on these results, we speculate that MSM taken during intense bouts of exercise would reduce postexercise immunosuppression.

## Figures and Tables

**Table 1:** Descriptive characteristics of 40 men assigned to either placebo or MSM.

Variable	Placebo *n* = 20	MSM *n* = 20
Age (yrs)	25.5 ± 1.2	25.1 ± 1.6
Height (cm)	178.8 ± 1.5	176.5 ± 1.4
Weight (kg)	83.6 ± 2.4	84.6 ± 1.5
BMI (kg·m^−2^)	26.1 ± 0.6	27.2 ± 0.5
Knee extensor 1RM (kg)	147.2 ± 4.1	151.9 ± 5.6
Compliance to capsule intake (%)	96.2 ± 1.4	96.6 ± 1.0

Data are mean ± SEM.

No statistically significant differences noted (*p* > 0.05).

**Table 2:** Blood cytokine data of 40 men assigned to either placebo or MSM for four weeks.

	Baseline 1	Baseline 2	At 0 hrs	After 24 hrs	After 48 hrs	After 72 hrs
IL-6 (pg·mL^−1^) *Placebo*	10.3 ± 5.0	18.3 ± 7.2	16.8 ± 6.9	15.4 ± 6.3	19.1 ± 9.5	17.6 ± 11.2
IL-6 (pg·mL^−1^) *MSM*	14.4 ± 4.4	11.6 ± 2.9	16.0 ± 3.1	17.9 ± 4.5	13.7 ± 3.5	13.9 ± 3.5

IL-8 (pg·mL^−1^) *Placebo*	32.8 ± 10.0	39.7 ± 14.6	46.4 ± 15.9	30.7 ± 12.3	31.2 ± 13.7	41.3 ± 14.9
IL-8 (pg·mL^−1^) *MSM*	28.3 ± 6.1	24.0 ± 5.4	37.6 ± 8.3	30.2 ± 5.6	24.5 ± 4.4	29.6 ± 7.2

IL-10 (pg·mL^−1^)^*∗*^ *Placebo*	6.1 ± 1.6	6.5 ± 1.7	5.7 ± 1.2	6.2 ± 1.5	4.8 ± 1.1	6.0 ± 1.5
IL-10 (pg·mL^−1^) *MSM*	22.6 ± 8.1	17.7 ± 6.6	26.8 ± 9.4	19.3 ± 6.8	18.4 ± 7.0	18.1 ± 6.9

IL-17 (pg·mL^−1^)^*∗*^ *Placebo*	54.3 ± 15.3	65.7 ± 22.2	72.9 ± 26.3	42.6 ± 13.6	47.2 ± 16.8	69.5 ± 25.1
IL-17 (pg·mL^−1^) *MSM*	27.3 ± 8.4	24.4 ± 7.2	33.5 ± 8.2	30.1 ± 7.7	22.8 ± 5.0	28.5 ± 6.9

IL-1*β* (pg·mL^−1^)^*∗*^ *Placebo*	3.7 ± 1.1	2.7 ± 0.8	2.8 ± 0.8	2.8 ± 0.7	3.3 ± 1.1	3.3 ± 1.0
IL-1*β* (pg·mL^−1^) *MSM*	10.8 ± 3.2	10.4 ± 3.2	15.5 ± 4.6	18.7 ± 7.6	15.8 ± 5.8	12.0 ± 3.4

TNF-*α* (pg·mL^−1^)^*∗*^ *Placebo*	3.3 ± 0.8	2.1 ± 0.4	3.4 ± 0.6	2.3 ± 0.7	2.3 ± 0.6	2.7 ± 0.8
TNF-*α* (pg·mL^−1^) *MSM*	4.8 ± 0.6	3.8 ± 0.5	5.9 ± 0.6	4.7 ± 0.7	4.1 ± 0.5	4.6 ± 0.7

Values are mean ± SEM.

^*∗*^Condition effect for IL-10 (*p* = 0.001), IL-17 (*p* = 0.0005), IL-1*β* (*p* = 0.0003), and TNF-*α* (*p* < 0.0001).

No other statistically significant effects noted (*p* > 0.05).
